# A closer look at discordant placental echogenicity: two cases under the microscope

**DOI:** 10.1002/ccr3.4506

**Published:** 2021-07-21

**Authors:** Mariano M. Lanna, Valentina Toto, Stefano Faiola, Daniela Casati, Gaetano P. Bulfamante, Irene Cetin, Maria Angela Rustico

**Affiliations:** ^1^ Fetal Therapy Unit Umberto Nicolini Department of Woman, Mother and Neonate Vittore Buzzi Children’s Hospital ASST Fatebenefratelli Sacco University Hospital Milano Italy; ^2^ Unit of Human Pathology Department of Health Sciences San Paolo Hospital Medical School University of Milano Milano Italy

**Keywords:** obstetrics and gynecology

## Abstract

Discordant placental echogenicity is observed in MC pregnancies complicated with twin anemia‐polycythemia sequence, but could also belong to complicated singleton gestation.

## Graphical abstract

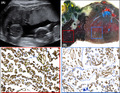

## 1 CASE SERIES

Histopahological comparison of two placenta with discordant echogenicity: a monochorionic with spontaneous twin anemia‐polycythemia sequence showed villi with continuous trophoblastic cover and increased fibrous stromal core in the anemic side; a singleton with intrauterine growth restriction and thrombosis of one umbilical artery with massive thrombotic vasculopathy in the hyperechogenic portion.

### 1.1 Case 1

**FIGURE 1:**
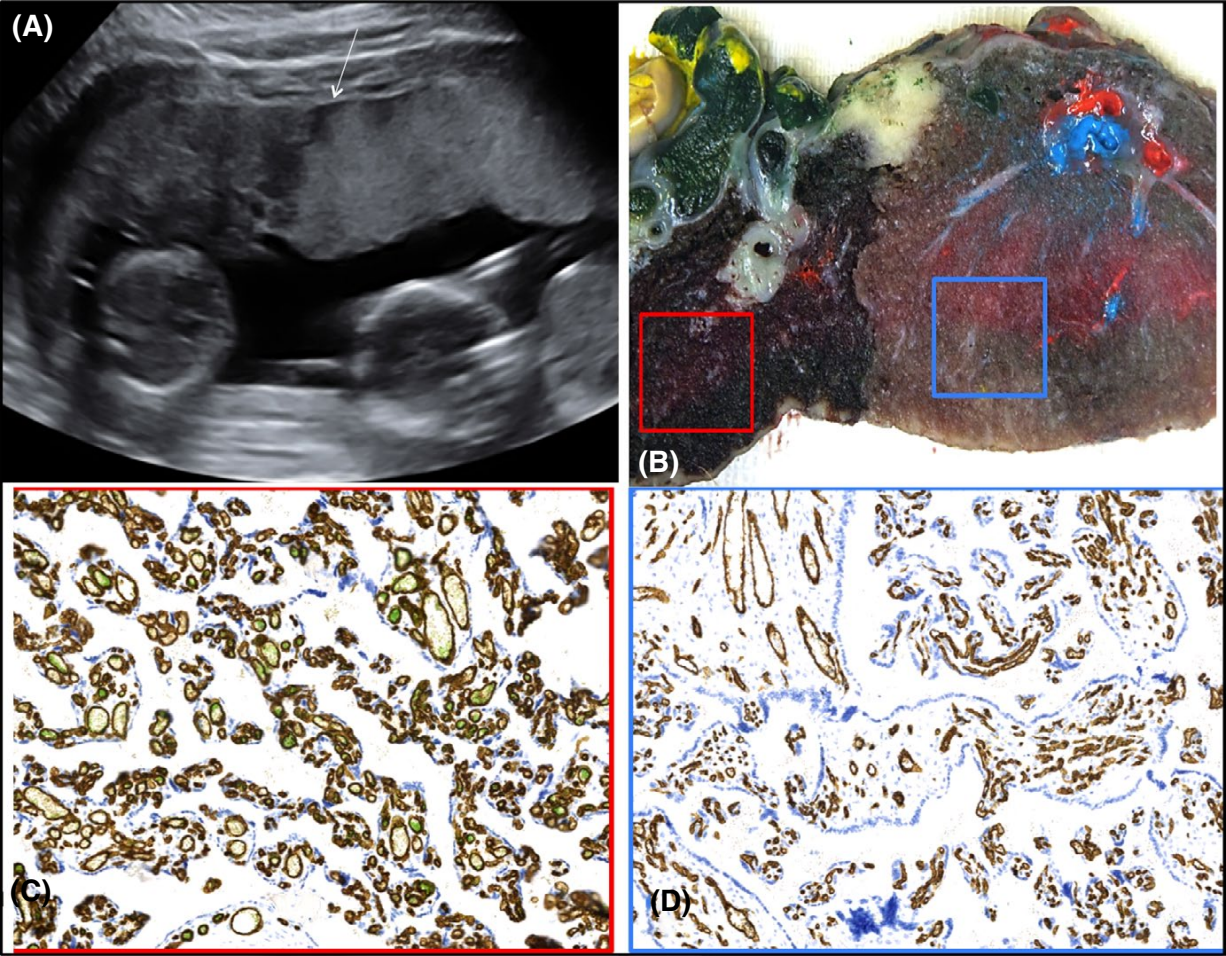
Case 1: Placenta of a monochorionic twin pregnancy complicated with TAPS. (A) Discordant placental echogenicity at 21.6 weeks. The hyperechogenic side (right of the arrow) belonged to the anemic twin, while the hypoechogenic side (left of the arrow) belonged to the polycythemic co‐twin. (B) Placental cut surface at the level of the vascular equator showing pale, light red areas corresponding to the anemic twin side, and dark red congested areas corresponding to the polycythemic twin side; (C) CD34 immunohistochemistry of the polycythemic twin placental side (corresponding to the red box in B) showing high prevalence of richly capillarized terminal villi (D) CD34 immunohistochemistry of the anemic twin placenta side (corresponding to the blue box in B), with high prevalence of stem and mature intermediate villi with continuous trophoblastic cover, increased fibrous stromal core and poorly developed fetal capillaries. (10x magnification)

**FIGURE 2:**
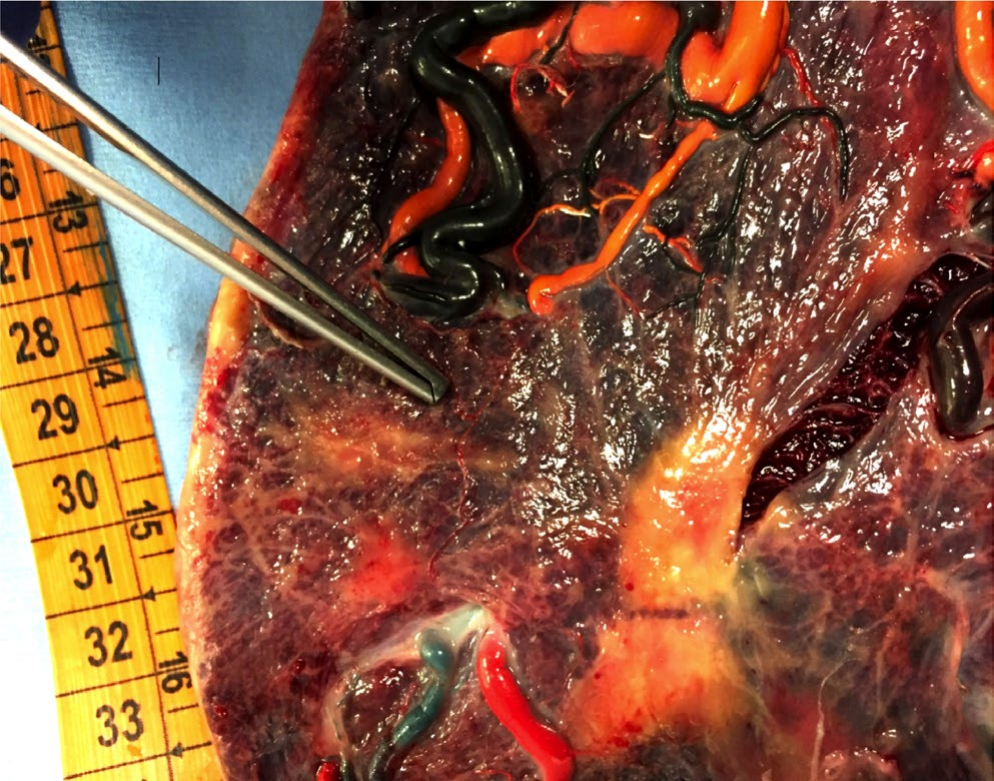
Case 1 Color dye placenta showing the minuscole artero‐venous anastomosis (between the blue arterial vessel and the green venous vessel)

A 30‐year‐old primigravida with twin gestation was referred at 15.5 weeks for the specific surveillance of monochorionic (MC) twin pregnancy. Middle cerebral artery peak systolic velocity (MCA‐PSV) was 43 cm/s (>1.5 MoM) in one twin, and 18 cm/s (1.00 MoM) in the co‐twin. This discrepancy, a preliminary indication of spontaneous twin anemia‐polycythemia sequence (TAPS), persisted at the following weekly ultrasound examinations. At 21.6 weeks, an obvious discordant placental echogenicity was detected (Figure 1). The hyperechogenic side belonged to the anemic twin (MCA‐PSV 43 cm/s, >1.5 MoM) and the hypoechogenic portion to the polycythemic co‐twin (MCA‐PSV 18 cm/s, <0.8 MoM). TAPS remained stable at Stage 1 without progression to more severe stages and was managed expectantly. The discordancy in placental echogenicity persisted throughout gestation although it was less marked after 28 weeks. A cesarean section was performed at 33.4 weeks, when MCA‐PSV was 78 cm/s (>1.5 MoM) and 20 cm/s (<0.8 MoM) in the anemic and polycythemic twin, respectively. The anemic neonate weighed 1490 g, had hemoglobin 6 g/dl, and reticulocyte counts of 13%; the polycythemic twin, weighing 1800 g, had hemoglobin of 24 g/dl, and reticulocyte counts of 2.4%. The two little girls are presently in good health at one year follow‐up. When color‐dye injection was performed to study the placental angioarchitecture, one very small artero‐venous anastomosis (diameter 1 mm) was found (Figure 2). The macroscopic and histological features of this TAPS placenta are described in detail in Figure 1.

### 1.2 Case 2

**FIGURE 3:**
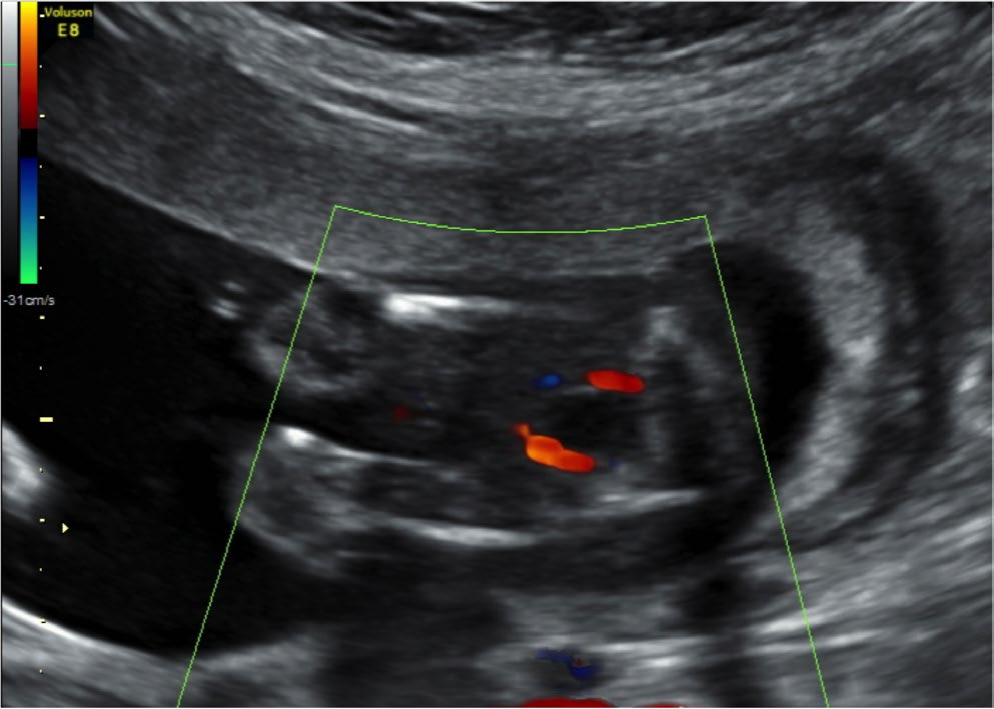
Case 2 evidence of two umbilical artery at 16 weeks gestation

**FIGURE 4:**
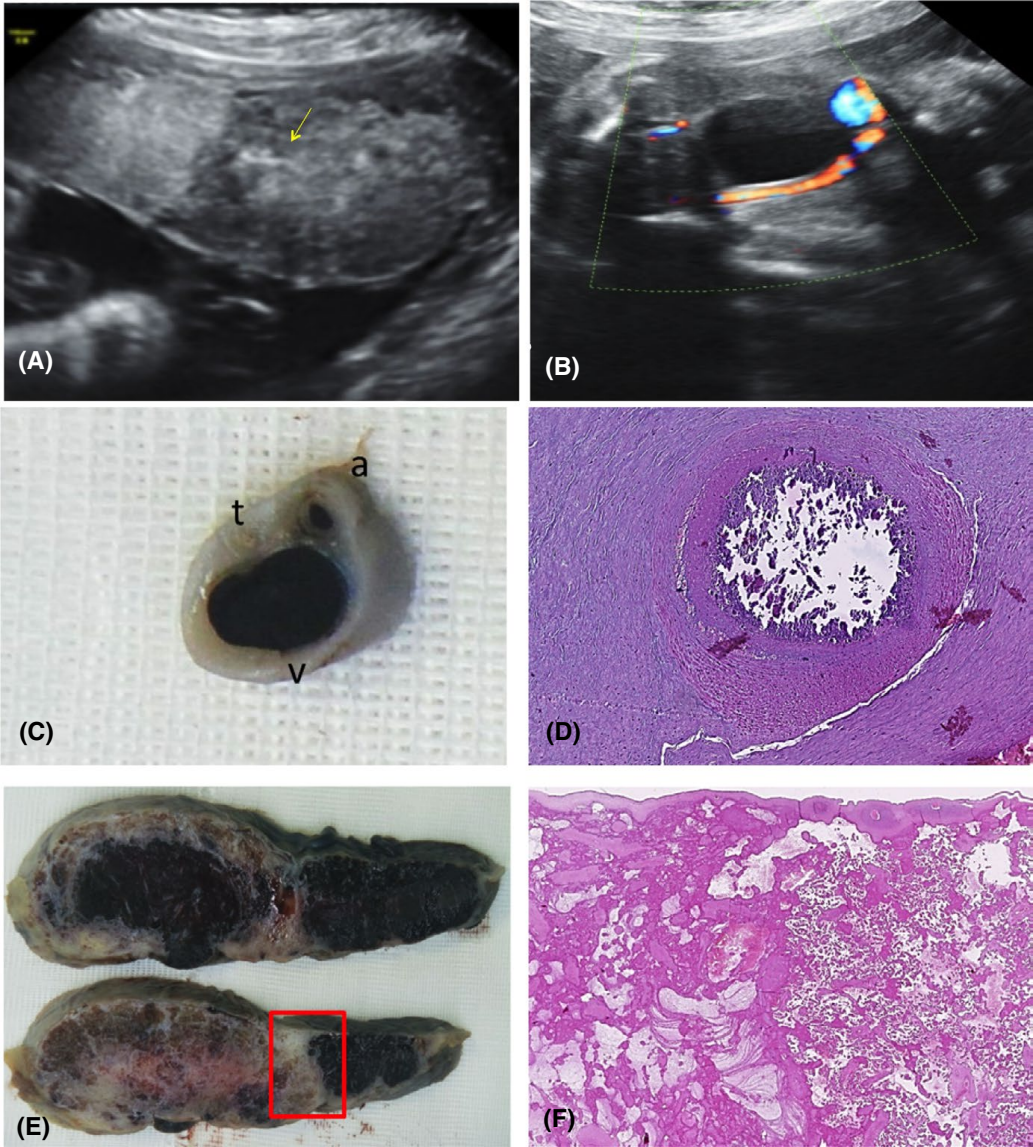
Case 2: Placenta of a singleton IUGR fetus complicated with thrombosis of one umbilical artery. (A) placental discordant echogenicity at 31.4 weeks. The hyperechogenic portion to the left of the arrow; (B) color Doppler image at the level of the fetal bladder, showing a single umbilical artery; (C) section through the umbilical cord: the thrombotic artery (t) has a very small diameter with a thickened wall; v, vein; a, artery. (D) microscopic appearance of calcification within the thrombus, (E) sharp border between pale, non‐perfused and dark red, well‐perfused placental parenchyma; (F) microscopic appearance of the interface between avascular and ischemic villi and normal premature villi (corresponding to the red box in E)

**FIGURE 5:**
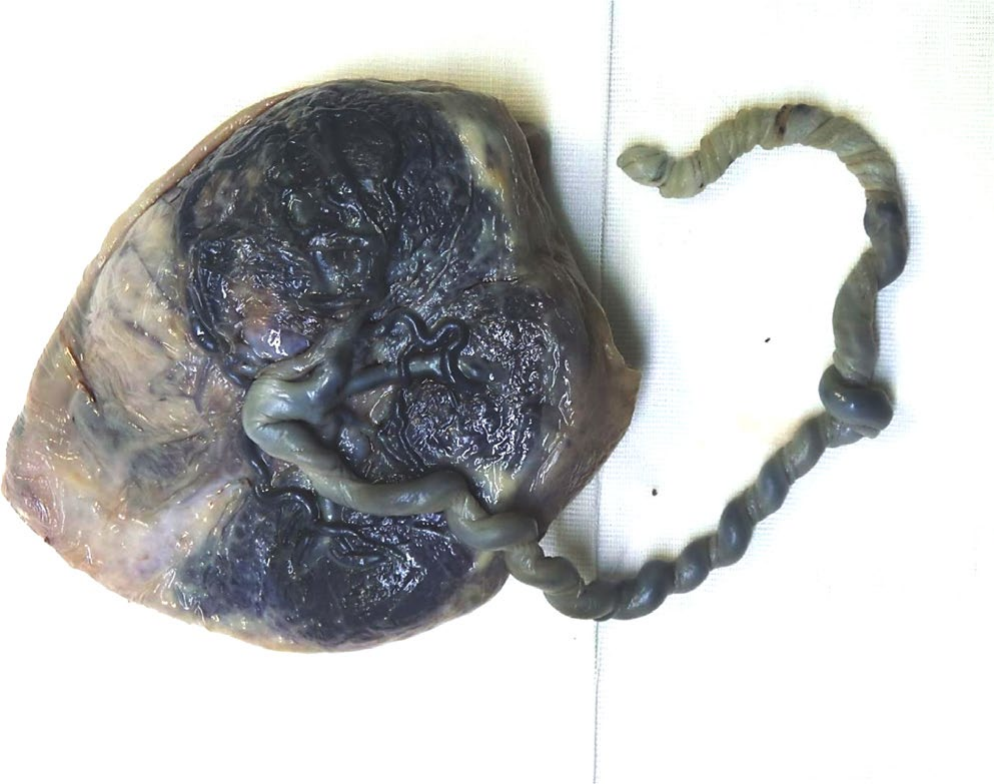
Case 2 Macroscopic image of the placenta, showing a highly twisted umbilical cord and a pale, white area (8x11cm) with massive reduction in the number and caliper of chorionic vessels

A 35‐year‐old woman in her first pregnancy, without any personal or family history, was referred for a second opinion at 31.4 weeks gestation because of unexplained intrauterine growth restriction (IUGR) first detected at 24 weeks gestation, in association with oligoanhydramnios which resolved 2 weeks later. Previous ultrasound examinations at 12, 16, and 20 weeks were normal. Karyotype was 46,xx, and TORCH serology was negative. Color Doppler images documenting the presence of two umbilical arteries (UA) were available at 16 weeks gestation (Figure 3). Ultrasound examination at referral showed a fetus growing below the 5^th^ centile, without structural anomalies. The placenta displayed an unusual discordant echogenicity: one‐third appeared hyperechogenic, while there was normal echogenicity in the remaining part (Figure 4). Fetal and maternal Doppler evaluation were normal. A single UA surrounding the fetal bladder was detected (Figure 4), suggesting the hypothesis of spontaneous UA thrombosis leading to IUGR. The placental feature remained substantially unchanged until 38 weeks, when a healthy baby girl weighing 2300 g was born spontaneously. Gross examination of the placenta (Figure 5) revealed a highly twisted umbilical cord (9 twists every 4 cm) with central insertion and three umbilical vessels with complete obliteration of one artery. The fetal surface had a pale white area measuring 8 × 11 cm, with a massive reduction in the number and caliper of chorionic vessels. The cut surface showed normal well‐perfused, dark red parenchyma in about 70% and a pale dark brown/white part of firmer consistency in about 30%. There was a clear demarcation between the two areas and in the transition point, the firmer part had an irregular “C‐shaped” appearance (Figure 4). Macroscopic and microscopic examination revealed umbilical artery thrombosis along the entire length of the umbilical cord. Calcifications were found intermittently within the thrombus, especially near the placental part (Figure 4). Microscopically, the fetal plate showed micro‐calcification in the occluded chorionic vessels, while the corresponding placental parenchyma displayed a massive old villous infarct associated with intervillous laminated hemorrhage. This kind of widespread fetal thrombotic vasculopathy (FTV) is the result of hemodynamic changes in both the feto‐placental and intravillous circulations. The non‐affected artery was well‐perfused, but the corresponding parenchyma displayed diffuse delayed maturation of the chorionic tree and focal Tenney‐Parker changes (Figure 4).

## 2 DISCUSSION

Discordant placental echogenicity is a sonographic finding first reported in monochorionic (MC) twin pregnancies complicated with severe TTTS.^1^ The same placental appearance has been observed in cases with TAPS, a recently described form of chronic feto‐fetal transfusion of erythrocytes.^2^, ^3^ Preliminary studies have found that this ultrasound placental appearance correlates with MCA‐PSV, and may assist in the prenatal identification and severity of TAPS.^4^, ^5^ Despite growing interest in the topic, few reports have described the histopathologic features of TAPS placentas with prenatally discordant echogenicity. Moreover, the two cases described show that discordant placental echogenicity is not peculiar to MC complicated twin pregnancies, but can also occur in complicated singleton pregnancies.

In TAPS placentas, it has been suggested that both the degree and duration of changes in erythrocyte levels play a role in causing this discordancy.^6^ Other authors ^3^ have observed increased erythroblastosis and small collapsed capillaries on the hyperechogenic placental side, and marked congestion of the villous capillaries on the polycythemic side. Another report described delayed villous maturation in the anemic placental side.^7^ We suggest that the hyperechogenicity of the anemic twin placental side might be connected to a widespread collapse of villous capillaries accompanied by stromal hyalinization in a globally immature villous tree, while the hypoechogenic side of the polycythemic twin might be associated with richly capillarized terminal villi. The histopathologic appearance could be consistent with a chronic fetal anemic condition, given that shortage of oxygen and nutrient compounds can lead to a delayed villous maturation and to an intravillous capillary collapse.

Umbilical artery thrombosis is a rare condition at high risk of fetal morbidity and mortality, which has been reported in association with highly twisted umbilical cord.^8^, ^9^, ^10^ Discordant placental echogenicity has never been reported in these cases. The massive FTV involving about 30% of the placental parenchyma was the origin of the discordant echogenicity observed. FTV is characterized either by the absence or degeneration of fetal capillaries in groups of contiguous terminal villi with a distribution consistent with upstream vascular occlusion.^11^ In this case, the sequence of events linking highly twisted umbilical cord to umbilical artery thrombosis might be attributed to hemodynamic alteration and thus endothelial damage, leading eventually to the vascular degeneration of large portions of the villous tree.

In conclusion, discordant placental echogenicity should be regarded as an indicator of potentially critical fetal conditions not only in MC twin pregnancies, but also in singletons, and demands dedicated surveillance and appropriate work‐ups.

## CONFLICTS OF INTEREST

The authors report no conflicts of interest or financial support.

## AUTHOR CONTRIBUTIONS

Mariano M. Lanna collected data and wrote the article. Valentina Toto and Gaetano P. Bulfamante performed histopathological exams. Stefano Faiola and Daniela Casati collected data. Irene Cetin reviewed the article. Maria Angela Rustico collected data, ideated, wrote, and reviewed the article.

## ETHICAL APPROVAL

Informed written consent was obtained from the patients. Due to retrospective nature of the study no approval from local ethical committee.

## CONSENT STATEMENT

Informed written consent was obtained from the patients.

## Data Availability

The data that support the findings of this study are available from the corresponding author upon reasonable request.
